# Functional properties of *Ditaxis heterantha* proteins

**DOI:** 10.1002/fsn3.34

**Published:** 2013-05-09

**Authors:** Ma T. Espino‐Sevilla, Maria E. Jaramillo‐Flores, Rodolfo Hernández‐Gutiérrez, Juan C. Mateos‐Díaz, Hugo Espinosa‐Andrews, Ana P. Barba de la Rosa, Jose O. Rodiles‐López, Socorro Villanueva‐Rodríguez, Eugenia C. Lugo‐Cervantes

**Affiliations:** ^1^ Departamento de Ciencias Tecnológicas Centro Universitario la Ciénega Av. Universidad, Núm. 1115 47820 Ocotlán Jalisco México; ^2^ Unidad de Tecnología Alimentaria Centro de Investigación y Asistencia en Tecnología y Diseño del Estado de Jalisco A.C., Av. Normalistas 800 44270 Guadalajara México; ^3^ Departamento de Graduados e Investigación de Alimentos Escuela Nacional de Ciencias Biológicas (ENCB) Instituto Politécnico Nacional (IPN) Carpio y Plan de Ayala 11340 México, D.F México; ^4^ Instituto Potosino de Investigación Científica y Tecnológica (IPCYT) Camino a La Presa de San José 2055, Lomas 4a Sección 78216 San Luis Potosí México

**Keywords:** *Ditaxis heterantha*, Euphorbiaceae, functional properties, protein characterization

## Abstract

*Ditaxis heterantha* is a plant of the Euphorbiaceae family that grows in semiarid regions of Mexico. It produces yellow pigmented seeds that are used for coloring of foods. The seeds contain about 20% of proteins. Proteins of *D. heterantha* were extracted and fractionated on the basis of solubility. Three main protein fractions were obtained: glutelins, 488 ± 0.5; albumins, 229 ± 2; and total globulins, 160 ± 1 g/kg. The amino acid profile was evaluated for each fraction and protein isolated, where the protein isolate contains essential amino acids such as Val, Phe, Tyr, and Leu. A calorimetric study showed that globulins and glutelins have a high denaturing temperature between 100 and 106°C, while albumins showed a denaturing temperature at 76°C. The protein isolate and its fractions exhibited functional properties: the isolated protein demonstrated good oil‐holding capacity of 40.7 g/kg. Foam capacity (FC) and foam stability (FS) were observed principally in glutelins and globulins where FC maximum was 330% and the FS was 28 min. The emulsifying capacity was observed in the same fractions of glutelins and globulins, followed by albumins. However, the glutelin fraction in particular was the only fraction that exhibited emulsifying stability at pH 5, 6, and 7. Gelling capacity was observed in albumins and globulins. This study indicated that protein isolated from *D. heterantha* could be used in food formulations due to its essential amino acid profile. Glutelin could be used as an emulsifying additive. Additionally, glutelin and globulin were stable at temperatures above 100°C; this is an important factor in food industry, principally in heat processes.

## Introduction

Proteins have become important ingredients in the food industry, enriching and improving the quality of foodstuffs and more recently, due to their functional properties that supply certain specific and attractive characteristics to the final product. Protein from seeds might possess desirable functional properties and provide essential amino acids for utilization in different food systems. In the food industry, the seeds most frequently used are soybean, pea, sunflower, and some cereals, due to their nutritional value and their functional properties, such as emulsification, solubility, foaming properties, water and oil absorption capacities and gelling properties (Kinsella and Phillips 1989), which have wide‐ranging applications in meat, dairy and bakery, noodles, soups and beverages, as well as in nutritional supplements (Renkema et al. 2000; González‐Pérez et al. 2005a).

In recent years, new protein sources from seeds have been reported, showing good functional properties in bitter melon, *Ginkgo biloba*, tepary (albumins and globulins), guava (glutelins), and cowpea proteins; thus, these can be a potential ingredient in certain food applications and promise new alternatives in the food industry (Idouraine et al. 1991; Ragab et al. 2004; Bernardino‐Nicanor et al. 2005; Deng et al. 2011; Horax et al. 2011).

In countries with limited resources, seeds from nonconventional species are utilized as protein source or additives, dyes, etc. These species comprise an alternative when there is an insufficient protein supply. This justifies increasing research on nonconventional food resources, especially on plants with high protein content, such as those of the Euphorbiaceae family (Tchiegang et al. 2006).

*Ditaxis heterantha*, also known as *Azafrán de bolita*, is an endemic plant in Mexico that belongs to the Euphorbiaceae family (Webster 1994). Its endosperm is yellow and it is used as a spice to supply color, aroma, and flavor to foods in Mexico in the semiarid zones where it is cultivated (Martínez 1990). The seeds of this plant contain 200 g/kg proteins (Méndez‐Robles et al. 2004). Early studies realized on Ditaxis heterantha seeds were on plant distribution in Mexico, pigment identification and aroma compound production by enzymatic hydrolysis from the carotenoid oleoresin (Méndez‐Robles et al. 2004, 2006; Del Toro‐Sánchez et al. 2006). However, to our knowledge, there are no reports on the characteristics and properties of the storage proteins present in these seeds. The characterization and determination of their functional properties could increase the value of *D. heterantha* seeds as a food additive, thus expanding its market value.

The main purpose of this study was to provide information on the physicochemical characterization of *D. heterantha* and to evaluate the effect of pH on the functional properties of the protein isolate and on protein fractions from *D. heterantha* seeds, which suggest their possible use as food ingredients in the food industry.

## Materials and Methods

### Materials

*Ditaxis heterantha* seeds were obtained from a local market in Guadalajara, Jalisco, Mexico.

### Preparation of defatted seed meal

The seeds were manually dehulled, ground, and defatted by the Soxhlet extraction system (AOAC 1924), employing petroleum ether (10% w/v) for 24 h at room temperature. Defatted meal (DM) was air‐dried at room temperature and passed through a sieve to obtain a fine powder (100 μm particle size); the protein was then analyzed by the Kjeldahl (*N* × 6.25) method and stored at 4°C until further use.

### Chemical composition

Moisture, protein, fat, ashes, and carbohydrates were analyzed by official methods (AOAC 1924) for the whole seed flour (unshelled) and for the defatted seed meal.

### Protein fractionation and preparation of protein isolates from *D. heterantha* seeds

Fractions (albumins, globulin I, globulin II, and glutelins) were obtained by the method of Osborne (1924) and *D. heterantha* protein isolate (*Dh*PI) was obtained by isoelectric point as described by Salcedo‐Chávez et al. (2002), the total globulin (TG) isolate was extracted according to the method of Blagrove and Gillespie (1978) as described by Ribeiro et al. (2004) without ammonium sulfate precipitation. The yield of each fraction was expressed as grams of proteins with respect to proteins present in the seed.

### Protein solubility

The protein solubility (PS) of protein samples was determined by the method of Bera and Mukherjee (1989) at room temperature and at pH 3, 5, 7, and 9. One hundred milligrams of protein was dispersed in 10 mL of 0.1 mol/L phosphate buffer. The suspension was stirred for 30 min, avoiding foam formation, and centrifuged at 10,000 × g for 30 min at 21°C. The protein content of the supernatants was determined by the method of Bradford (1976).

The PS in each sample was calculated as follows:

$$\text{PS}\;(\%)=\frac{\text{Amount of protein in the supernatant}}{\text{Amount of protein in the sample}}\times 100.$$

### Gel electrophoresis (SDS‐PAGE)

Sodium dodecyl sulfate‐polyacrylamide gel electrophoresis (SDS‐PAGE) was performed according to the Laemmli method (Laemmli 1970) on a Mini‐PROTEAN II electrophoresis cell (Bio‐Rad, Hercules, CA). Protein reduction was performed by 2‐ME in 13% (w/v) polyacrylamide gels. After electrophoresis, the samples were stained with Coomassie brilliant blue. Low‐molecular‐weight markers, ranging from 14 to 97 kDa, were used as standard (Amersham, Pharmacia Biotech, Uppsala, Sweden).

### Amino acid analysis

The amino acid profile was measured by means of high‐performance liquid chromatography (HPLC) (Varian model 5000; Walnut Creek, CA)‐controlled microprocessor coupled to a fluorichrom fluorescence detector equipped with a deuterium lamp. Primary amino acid separation was performed on an HPLC RP‐C18, 3 μm column (25 cm × 4.6 mm). A Lichrosorb RP‐C18, 5 μm (30 cm × 4.6 mm ID) column, was used for the determination and separation of secondary amino acids (Alltech/Applied Science, PA). The amino acids were detected with the monochromator set at 330 nm and with a 418 nm cutoff filter according to Vázquez‐Ortiz et al. (1995). Duplicate runs were performed and mean values are reported.

### Differential scanning calorimetry (DSC)

Denaturation temperatures (*T*_d_) for *D. heterantha* proteins were investigated utilizing Q2000 TA‐Instrument differential scanning (New Castle, DE). Lyophilized samples of *D. heterantha* protein fractions (approximately 10–15 mg in size) were placed in hermetic steel pans and resuspended with 20 μL of the extraction buffer of each protein. The pans were sealed and stabilized at 20°C for 1 h prior to DSC analysis. A heating ramp at 10°C/min (40–150°C) was used. An empty pan was utilized as a reference. The DSC cell was purged employing a 50 mL/min flow of N_2_. *T*_d_ and *ΔH*_d_ were estimated employing Universal Analysis (TA Instruments).

### Functional properties

#### Water and oil‐holding capacity (WHC‐OHC)

Samples of *Dh*PI were measured by the method of Beuchat (1977). WHC was expressed as the volume in mL of water held by 1 g of protein sample. OHC was expressed as g of oil held by 1 g of protein sample (g/g) and the oil density was 0.921 g/mL.

#### Foam capacity (FC) and foam stability (FS)

Protein samples were determined by the method of Kato et al. (1983) as described by Rodiles‐López et al. (2008). Air (90 cm^3^/min) was introduced into a glass tube (2.4 × 30 cm) containing 10 mL of 1 g/kg protein in 0.1 M of phosphate buffer (pH 3, 5, 7, 9) for 15 s. The volume of foam in cm^3^ was measured immediately after switching off the gas flow by directly reading the dispersion height in the glass tube. FC was evaluated employing the formula of Wilde and Clark (1996) as follows:

$$\text{FC}\;(\%)=\frac{{\text{Volume foam (mL)}}_{\mathrm{with}\;\mathrm{air}}}{{\text{Volume sample (mL)}}_{\mathrm{with}\;\mathrm{air}}}\times 100.$$

Foam stability was determined by measuring the conductivity of the foams produced when air at 90 cm^3^/min constant flow rate was introduced into 20 mL of a protein solution (1 g/L) in 0.1 M phosphate buffer. The flow was maintained until the foam reached a volume of 38.42 cm^3^. Conductivity readings were taken every minute for 5 min, a plot of log_10_ (conductivity) versus time was constructed with a regression equation of the form

y=mx+b,

where *y* is log_10_ (conductivity) and *x* is time. All of the equations thus determined gave *R* ^2^ ≥ 0.98. FS was calculated from the following equation:

FS(min)=b/m.

The parameter *b* is equal to log (*C*_i_), where *C*_i_ is the initial conductivity or foaming power as defined by Kato et al. (1983).

#### Emulsifying capacity (EC) and ES

Emulsifying capacity and ES were evaluated by the turbidimetric technique reported by Pearce and Kinsella (1978). To a mixture of 30 mL of 5% protein solution in 0.1 mol/L phosphate buffer (pH 3, 5, 7, 9) was added 10 mL of corn oil. The mixture was homogenized for 1 min at 11,000 rpm with an Ultraturrax UAC 30‐R X‐520 homogenizer. Fifty milliliters of emulsion was dispensed into 5 mL of SDS solution (1 g/L) immediately and at 10 min after homogenization. Absorbance of the solution at 500 nm was measured with a UV–vis Spectrophotometer (CINTRA 6 GBS, Braeside, Vic.).

Emulsifying capacity was calculated as follows:

$$\text{EC}=\frac{2\mathrm{Tb}}{\Phi C},$$

where *C* is the weight of protein per unit volume of aqueous phase before the emulsion is formed and Φ is the volume fraction of the dispersed phase (0.667 in this experiment).

*T*_b_ is turbidity defined by Pearce and Kinsella (1978) as *T*_b_ = 2.303*A/l*, where *A* is the emulsion absorbance and *l* is the path length of the cuvette (1 cm).

ES was calculated as follows:

$$\text{ES}=\frac{T_{b0}\Delta t}{\Delta T_{b}},$$

where ∆*T*_b_ is the change in turbidity over time interval ∆*t* and *T*_b0_ is the initial turbidity of the sample.

#### Gelling capacity

Least gelation concentration (LGC) of protein was measured according to Coffmann and García (1977). LGC was determined as the concentration (g/L) when the sample from the inverted test tube did not fall or slip.

### Statistical analysis

All analyses were performed in triplicate and expressed as mean values ± standard deviation (SD). Data were analyzed using one‐way analysis of variance. A 95% confidence level was applied. Comparison of means was performed by the least significant difference (Tukey) test at a significant *P*‐value level of 0.05.

## Results and Discussion

### Chemical composition of *D. heterantha* meal

The chemical composition of *D. heterantha* whole meal and DM is shown in Table 1. The protein content of *D. heterantha* was found to be 262.3 ± 0.36 and DM, 609.3 ± 1.45 g/kg. This protein content is similar to other sources studied of the same Euphorbiaceae family, such as *Ricinodendrum heudelotii*, with 200 g/kg protein, respectively, for whole meal (Tchiegang et al. 2006). In addition to comparison with other sources utilized in the food industry, the protein content of *D. heterantha* was found to be higher than amaranth (150–180 g/kg) and sunflower proteins (200 g/kg) (Barba de la Rosa et al. 1992; González‐Pérez et al. 2005b).

**Table 1:** Chemical composition of whole and defatted meal from *Ditaxis heterantha* seed (g/kg)

	Meal	
Analysis	Whole	Defatted
Moisture	41.8 (±2.03)	86.0 (±0.92)
Fat	458.7 (±2.07)	4.7 (±0.15)
Protein	262.3 (±0.36)	601.3 (±1.45)
Crude fiber	186.4 (±1.60)	67.5 (±2.40)
Carbohydrates	9.2 (±0.15)	287.5 (±0.14)
Ash	28.2 (±0.26)	65.7 (±0.57)

All values are means (±) standard deviation of triplicate analyses.

### Protein fractionation

Protein fractionation performed by the Osborne method on the basis of solubility showed that glutelin was the main fraction, with 488 ± 0.5 g/kg, followed by albumin and globulin II, with yields of 229 ± 2 and 96 ± 2 g/kg of crude protein, respectively. Globulin I contributed 36 ± 1 g/kg and prolamin was present at negligible levels of 3.7 ± 0.3 g/kg (Table 2). Previous works conducted in seeds of the same family have reported a protein content of ~250 g/kg for albumins in *Plukenetia volubilis* seed (Shate et al. 2002) and protein contents of 444, 398, 123, and 340 g/kg for globulins, glutelins, albumins, and prolamins, respectively, for *Jatropha curcas* (López‐Laredo et al. 2005). These patterns were similar for albumins and glutelins from *D. heterantha*. The yield of TG extracted by the Blagrove and Gillespie (1978) method was 180 g/kg, which was higher than the yield obtained for globulin I + II of 130 g/kg by the Osborne method. These results can be due to the presence of Ca^2+^ and Mg^2+^ cations and chelating agents such as ethylene diamine tetraacetic acid (EDTA) and ethylene glycol tetraacetic acid (EGTA) during extraction, which increases the extraction efficiency of this protein (Freitas et al. 2000). This yield of TG (160 g/kg) is comparable to the yield of amaranth (200 g/kg) and soybean globulins (230 g/kg) (Gorinstein et al. 2001). The protein extraction method for DM at pH 11 and at a precipitation at pH 4.5 allowed to obtain a greater yield of 470 g/kg of *Dh*PI. This value was slightly lower than that obtained for amaranth and soy. This could be due to the fact that not all proteins in the seed have the same isoelectric point, leaving unprecipitated proteins, thus explaining the difference in the extraction efficiency.

**Table 2:** Yield of the isolate, fractionation procedure, and protein content of *Ditaxis heterantha* products

		Yield (g/kg)	
	Protein content^a^	Solids^b^	Protein^c^
SM	260 ± 2.0	100	100
DM	600 ± 3.0	440 ± 2.0	980 ± 2.0
*Dh*PI	870 ± 2.0	140 ± 1.0	470 ± 2.0
TG	930 ± 1.0	200 ± 1.0	160 ± 1.0
Albumins	860 ± 3.0	286 ± 2.0	229 ± 2.0
Globulin I	540 ± 3.0	66 ± 1.4	36 ± 1.0
Globulin II	750 ± 2.0	128 ± 2.3	96 ± 2.0
Prolamins	820 ± 0.5	6.5 ± 0.3	3.7 ± 0.3
Glutelins	900 ± 2.0	542 ± 4.1	488 ± 0.5

SM, seed meal; DM, defatted meal; *Dh*PI, protein isolate from *Ditaxis heterantha*, TG, total globulin and Osborne fractions (albumins, globulin I, globulin II, prolamins, and glutelins).

a Expressed as g/kg of protein in the *D. heterantha* protein product.

b Expressed as grams of solids with respect to the amount present in the seed.

c Expressed as grams of proteins with respect to the proteins present in the seed.

### Solubility

**Figure 1:**
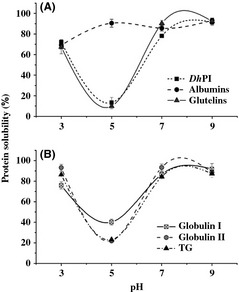
Effects of pH on the solubility of *Ditaxis heterantha* proteins. (A) *Ditaxis heterantha* protein isolate (*Dh*PI), albumins, and glutelin fractions. (B) Globulin I, globulin II, and total globulin (TG) fractions. Error bars: Standard deviation results are the means of determinations in triplicate.

The solubility of each fraction and *Dh*PI is depicted in Figure 1. *Dh*PI exhibited maximum solubility at pH 9 (91 ± 3.0%). However, the fractionated protein exhibited a different behavior: for example, the albumin fraction remained soluble independently of the pH, demonstrating a solubility range of 69.2–93.2% and did not exhibit a U‐shaped behavior. This behavior type has been observed in albumins from common buckwheat and sunflowers seeds, in which solubility remained >60% (González‐Pérez et al. 2005b; Tan and Wang 2010). These data suggest that the albumin fraction would possess good potential for use at different pH. Glutelin fraction and *Dh*PI showed similar behavior at pH 3, 5, and 9 (Fig. 1A), while on the other hand, globulin II showed a solubility of 93.0 ± 3.3 at pH 3, of 20.0 ± 1.3 at pH 5, of 91.3 ± 3.3 at pH 7, and of 88.5 ± 1.4% at pH 9 (Fig. 1B). These results were similar to TG behavior under the same pH conditions. Globulin I exhibited a slightly different behavior at pH 5, with minimum solubility of ~40%. The behavior of globulin I was similar to that of cowpea vicilin (7S globulin), with values >90% at pH 9 and of 80% at pH 4. The similar behavior between TG and globulin II can be explained from the observation that globulin II is composed of nearly 90% of TG, and the solubility of the former was higher than that of globulin from canola at pH 3, 7, and 9, with solubilities of 81, 60, and 80%, respectively (Lawal et al. 2005). When comparing TG with soy protein, TG showed a higher solubility than soy, with the solubility of soybean protein being 67.7, 61.8, and 66.2% at pH 3, 5, and 7, respectively (Idouraine et al. 1991; Marcone and Kakuda 1999). Glutelin fraction from guava extracted by a similar method exhibited a similar behavior at pH 3 and 9, with values of 64 and 95.9%, respectively (Bernardino‐Nicanor et al. 2005).

All protein fractions and the *Dh*PI, except the albumin fractions, exhibited a U‐shaped solubility curve. This is due to the net positive and negative charges acquired by the protein, which promote repulsion of the molecules and increase solubility, an important factor in properties such as emulsification, foaming, and gelling (Seena and Sridhar 2005). This solubility pattern was similar to that of other Euphorbiaceae, such as *R. heudelotii* (Bail), *T. conophorum* (Tchiegang et al. 2006), and *J. curcas* and *D. heterantha* in DM (Lestari et al. 2011).

### Sodium dodecyl sulfate‐polyacrylamide gel electrophoresis (SDS‐PAGE)

**Figure 2:**
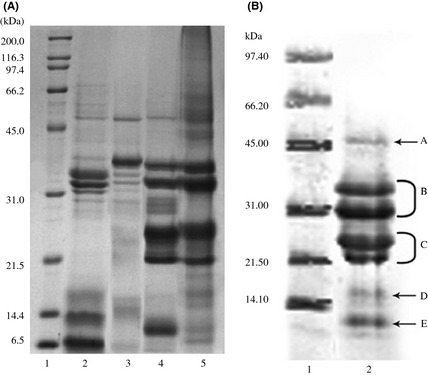
Electrophoretic pattern of protein fractions from *Ditaxis heterantha* under reduced conditions. (A) Osborne protein fractions: albumins (lane 2), globulin I (lane 3), globulin II (lane 4), and glutelins (lane 5). (B) Total globulin fraction obtained by the method of Blagrove and Gillespie (lane 2). Lane 1 in both figures represents standard protein. Molecular band weights are denoted by uppercase letters.

SDS‐PAGE of protein fractions from *D. heterantha* is illustrated in Figure 2. Albumins showed two principal bands of molecular weight (MW) polypeptides: one triplet of 31, 32, and 35 kDa and other band subunits of 8–14 kDa (Fig. 2A, lane 2), a typical characteristic of 2S albumins (Shewry and Pandya 1999). These results were similar to those for soybean and amaranth albumins, with MW of 34.2 and 36.4 kDa, respectively (Gorinstein et al. 2001). Barba de la Rosa et al. (1992) reported a band of 34 kDa, as well as several minor bands within the range of 14–28 kDa for amaranth albumins. On the other hand, sunflower albumins showed two main bands with MW of 12 and 14 kDa (González‐Pérez et al. 2005b). Globulin fractions (I and II) exhibited polypeptide bands of 50, 40, 36, and 28 kDa and one broad but weaker band between 16 and 18 kDa for globulin I (Fig. 2B, lanes 3 and 4). These MW were similar to those of pea and cowpea vicilin, indicating these are a typical characteristic of 7S globulin, in agreement with data previously reported by several groups (Rangel et al. 2003). TG (Fig. 2B, lane 2) showed two pairs of high‐intensity bands (B and C) that corresponded to polypeptides of 27–35 and 18–25 kDa, respectively (Fig. 2B, lane 2) and a weak band of 10 kDa (E), which may correspond to type II globulins, whose characteristic is to possess acidic (B) and basic (C) polypeptides, respectively, which is characteristic of the 11S globulin type (Shewry and Pandya 1999). Two MW bands, of 43 kDa (A) and 14 kDa (D), were observed and could correspond to globulin I bands (Fig. 2A, lane 3).

Similar observations were reported by Barba de la Rosa et al. (1992) for amaranth in type II globulins with bands in the range of 23–33 kDa and by Mo et al. (2006) who reported isolates of soybean glycinin acid subunits of MW in the range of 36–38 kDa and basic subunits of 20–22 kDa. Barba de la Rosa et al. (1992) described two polypeptides of 14 and 43 kDa, which could correspond to globulin II in amaranth. Finally, the glutelin fraction (Fig. 2A, lane 5) showed four polypeptides with intensive bands of 18, 30, 33, and 35 kDa.

### Amino acid analysis

The amino acid composition of each protein fraction and of *Dh*PI is presented in Table 3. In terms of essential amino acids, the amino acids that predominate in the seed are isoleucine, which is present in *Dh*PI, and globulin II with 53.5 ± 0.08 and 45.9 ± 0.07 g/kg, respectively, leucine in *Dh*PI, globulin II, and glutelin with 74.3 ± 0.02, 68 ± 0.03, and 59 ± 0.05 g/kg, respectively, and Phe + Tyr in all proteins except albumin. These values indicate that nutritionally, *Dh*PI*,* globulin II, and glutelin contain essential amino acids in amounts recommended by the FAO/WHO/UNU (1985) for children and adults.

**Table 3:** Amino acid composition (g/kg) of protein fractions from *Ditaxis heterantha* seed

Amino acid	g of amino acid/kg of crude protein						
	Protein					FAO/WHO/UNU reference pattern	
	*Dh*PI isolate	Albumins	Globulin I	Globulin II	Glutelins	Preschool child^a^	Adult^b^
Essential							
Isoleucine	53.5 ± 0.08	24.1 ± 0.02	28.1 ± 0.08	45.9 ± 0.07	36.0 ± 0.02	36	25
Leucine	74.3 ± 0.02	46.9 ± 0.05	44.0 ± 0.02	68.0 ± 0.03	59.0 ± 0.05	52	47
Lysine	38.9 ± 0.06	49.8 ± 0.03	54.9 ± 0.05	20.6 ± 0.02	26.0 ± 0.01	63	25
Methionine	18.7 ± 0.04	21.1 ± 0.02	12.3 ± 0.08	14.3 ± 0.05	14.7 ± 0.03	25^d^	25^d^
Phenylalanine + tyrosine	71.0 ± 0.04	27.9 ± 0.06	50.7 ± 0.04	72.4 ± 0.06	53.9 ± 0.03	52^e^	47^e^
Threonine	28.3 ± 0.02	11.8 ± 0.02	10.5 ± 0.02	24.0 ± 0.01	18.9 ± 0.04	43	27
Valine	65.8 ± 0.01	26.0 ± 0.01	31.0 ± 0.02	56.0 ± 0.02	39.5 ± 0.07	40	40
Histidine	28.3 ± 0.02	12.2 ± 0.02	25.4 ± 0.06	24.1 ± 0.08	19.5 ± 0.03	19	18
Nonessential							
Alanine	52.9 ± 0.09	29.2 ± 0.01	30.7 ± 0.03	49.5 ± 0.05	40.5 ± 0.06	ND	
Arginine^c^	130 ± 0.05	86.3 ± 0.01	56.7 ± 0.01	129 ± 0.02	88.7 ± 0.04	ND	
Glycine	60.9 ± 0.02	40.4 ± 0.05	233.7 ± 0.06	53.2 ± 0.01	102.1 ± 0.05	ND	
Aspartic acid	104 ± 0.04	38.9 ± 0.07	49.6 ± 0.07	88.2 ± 0.08	58.3 ± 0.09	ND	
Serine	35.2 ± 0.07	15.6 ± 0.02	47.1 ± 0.03	34.4 ± 0.04	22.5 ± 0.04	ND	
Glutamic acid	192 ± 0.04	131 ± 0.08	107.8 ± 0.02	157 ± 0.05	125.4 ± 0.02	ND	
Total	954.50	561.50	782.50	836.00	705.00		
Essential amino acid	378.80	219.80	256.90	325.30	267.50		
E/T (%)	39.69	39.15	32.83	38.91	37.94		

Values are expressed as mean ± standard deviation. ND, not determined.

a FAO/WHO/UNU Expert Consultation (2004). Report on Human Energy Requirements. Interim Report. Rome: FAO.

b Food and Nutrition Board/Institute of Medicine (2002). Dietary Reference Intakes (DRI) and Recommended Dietary Allowances (RDA) for energy, carbohydrate, fiber, fats, fatty acids, cholesterol, proteins, and amino acids. Institute of Medicine of the National Academies. Washington, DC: The National Academies Press.

c In some cases is considered as essential amino acid for children.

d Requirements for methionine + cysteine.

e Requirements for phenylalanine + tyrosine.

On the other hand, amino acids such as glutamic acid, aspartic acid, and arginine were present in significant amounts in all fractions and the protein isolate, which may contribute to functional properties such as EC.

### Differential scanning calorimetry

**Figure 3:**
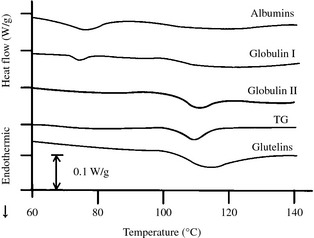
Differential scanning calorimetry thermograms of protein fractions from *Ditaxis heterantha*. TG, total globulin.

DSC thermograms of *D. heterantha* proteins are depicted in Figure 3. The DSC technique permits thermally induced unfolding of protein molecules to be detected and to determine denaturing temperature (*T*_d_) and enthalpy (*ΔH*_d_). High *T*_d_ peaks of 105.4, 104.8, and 102.5°C and of 2.20, 4.47, and 2.08 W/g for globulin II, glutelin, and TG fractions, respectively, and peaks of 76.4 and 70.5°C and of 0.37 and 0.97 W/g for globulin I and albumin fractions, respectively, were observed. Martínez and Añón (1996) reported peaks of *T*_d_ in amaranth at 64°C for albumins and a peak of 96°C for glutelin and one of 94°C for 11S globulins. In recent studies, Condés et al. (2012) reported two characteristic endotherms: one at 70.7°C, which can be attributed to the denaturation of albumins and a minor globulin fraction (7S globulin), and a second endotherm at 98.6°C that corresponds to denaturation of the protein fractions of globulin 11S and glutelins for the amaranth isolate. This behavior was similar to that of *D. heterantha* proteins. The higher *T*_d_ shown by *D. heterantha* proteins indicates a thermostable character, which is commonly related to a higher number of hydrophobic interactions (Myers 1990).

### Functional properties

#### Water and oil‐holding capacity

WHC was evaluated in protein isolate from *D. heterantha* and was found to be 1.97 ± 0.03 mL. This value is lower than that obtained for the protein isolate from soybean (3.46 mL) (Chau et al. 1997). However, the WHC of *Dh*PI is higher than that obtained for the cowpea protein isolate (1.68 mL) (Mwanjala et al. 1999) and sesame protein isolate (1.5 mL) (Khalid et al. 2003), all of which have been widely studied. The results are within the range of WHC values for commercial protein concentrates (1.90–2.20 mL), as has been reported by Lin and Zayas (1987). Water holding by proteins is a function involving several parameters, such as size, shape, amino acid hydrophilic–hydrophobic balance in the protein molecule, compounds associated with the proteins and the physicochemical environment (pH, ionic strength, temperature, presence/absence of surfactants, etc.) (Shate and Salunkhe 1981).

#### Oil‐holding capacity

OHC was analyzed in *Dh*PI and showed 4.07 ± 0.06 g oil/g proteins. This value is higher than that of the ginkgo protein isolate (2.95 g/g), sesame protein isolate (1.5 g/g), soybean isolate (2.29 g/g), and those of pea and faba bean isolates, with values of 1.2 and 1.6 g/g, respectively (Khalid et al. 2003; Deng et al. 2011). A high OHC value indicates a major concentration of nonpolar residues. Kinsella (1976) established that with higher amounts of hydrophobic residues, the protein underwent more interactions with lipids. Soybean isolates are excellent emulsifiers and binders in high fat foods and this characteristic has been associated with their high OHC, as in pea and faba bean, with OHC of 0.98 and 1.79 g/g, respectively (Sosulski and McCurdy 1987). Therefore, *Dh*PI could be considered a potential food ingredient in fatty foods.

#### Foaming capacity (FC) and foaming stability (FS)

The effect of pH on FC and FS is presented in Table 4. For all samples studied, the values obtained ranged from ~286 to 377% (*P *<* *0.05) in which glutelins, *Dh*PI, and TG showed high FC values at pH 3, 7, and 9 and the best FS at 28 min. Only at pH 5, globulins I and II exhibited good FC and FS.

**Table 4:** Foaming capacity (FC) and foaming stability (FS) of protein from *Ditaxis heterantha* seed: albumins, globulin I, globulin II, glutelins, total globulins (TG), and *Ditaxis heterantha* protein isolate (*Dh*PI) at pH 3, 5, 7, and 9

Sample	pH 3		pH 5		pH 7		pH 9	
	FC (%)	FS (min)	FC (%)	FS (min)	FC (%)	FS (min)	FC (%)	FS (min)
Albumin	320 ± 16^a^	11 ± 0.4^a^	298 ± 16^a^	11 ± 0.4^a^	320 ± 16^a^	15 ± 0.4^b^	286 ± 16^a^	17 ± 0.3^c^
Globulin I	309 ± 32^a^	5 ± 1.1^a^	332 ± 32^a^	10 ± 0.5^b^	286 ± 32^a^	15 ± 0.4^c^	355 ± 0^a^	13 ± 0.4^c^
Globulin II	298 ± 16^a^	6 ± 0.3^a^	343 ± 16^b^	16 ± 0.3^b^	332 ± 0^ab^	32 ± 0.2^d^	341 ± 19^b^	28 ± 0.4^c^
Glutelin	355 ± 32^b^	22 ± 0.2^b^	286 ± 0^a^	ND^a^	343 ± 16^b^	26 ± 0.2^c^	332 ± 32^ab^	28 ± 0.2^d^
TG	377 ± 32^b^	28 ± 0.2^d^	320 ± 16^ab^	13 ± 0.4^a^	309 ± 16^a^	22 ± 0.2^c^	332 ± 0^ab^	17 ± 0.3^b^
*Dh*PI	332 ± 16^b^	28 ± 0.2^d^	298 ± 16^a^	6 ± 0.9^a^	332 ± 0^b^	15 ± 0.4^b^	309 ± 0^a^	25 ± 0.2^c^

Each value in the table is the mean of duplicate analyses ± SD. Means within a column followed by different letters are significantly different (*P *<* *0.05); ND, not detected.

Of all fractions studied, TG exhibited the highest FC at pH 3 (377 ± 32%) and at pH 7, a higher value was observed (309 ± 32%) than in globulin from tepary bean (150%) processed under similar conditions (Idouraine et al. 1991). Additionally, TG showed a FC of 320 ± 16% at pH 5 (near its isoelectric point), a behavior similar to that reported for amaranth globulins (Marcone and Kakuda 1999). The authors attributed this behavior to the lack of repulsive interactions near the isoelectric point, which would promote protein–protein interactions and the formation of viscous films at the air/water interface, in addition to increasing the amount of proteins absorbed.

The method employed in this work for evaluating FC has not been used in seed proteins and has only been utilized in α‐lactalbumin, an animal protein. We previously found that the FC for this protein was 286% at all pH profiles studied (3, 5, 7, and 9) (Rodiles‐López et al. 2008). Finally, *Dh*PI showed a higher FC (332 ± 0%) than that of the soy protein isolate (237%), sesame protein isolate (~90%), all of these at pH 7 processed under similar conditions (Blagrove and Gillespie 1978; Shewry and Pandya 1999), suggesting its possible application as a food ingredient.

#### Emulsifying capacity and emulsifying stability

**Figure 4:**
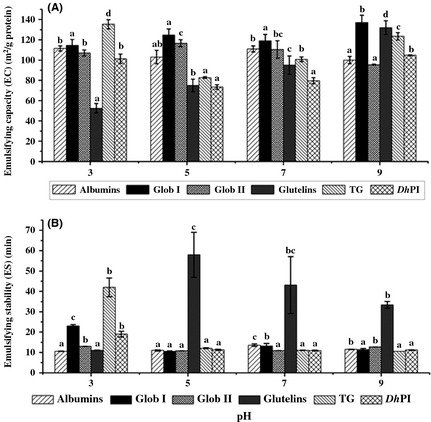
Effect of pH on the emulsifying capacity (EC) (A) and emulsifying stability (ES) (B) of proteins from *Ditaxis heterantha*: albumins, globulin I, globulin II, glutelins, total globulins (TG), and *D. heterantha* protein isolate (*Dh*PI) at pH 3, 5, 7, and 9. Error bars: Standard deviation results are means of determinations in triplicate. Bars with different letters have mean values that are significantly different (*P *<* *0.05).

The effect of pH on the EC of *D. heterantha* proteins is shown in Figure 4A. Globulin I and glutelins showed highest EC at pH 3 and 9, respectively.

The EC of glutelins increases as the pH increases, reaching its maximal EC at pH 9, starting from a 50 to 130 m^2^/g protein. This probably explains the soluble nature of glutelins at alkaline pH. Globulin I exhibited a maximum EC value of 137 ± 6 m^2^/g protein at pH 9, with the EC ranging from 110 to 137 m^2^/g protein between pH 3 and 9. Molina et al. (2001) reported an EC of 60 and ~180 m^2^/g protein for globulin 7S and 11S fractions from soybean, respectively, at pH 7, where 7S globulin had a very low value in comparison with globulin I (120 m^2^/g protein) from *D. heterantha*. Globulins II showed a low EC of 117 ± 3 m^2^/g protein on comparison with all fractions studied at pH 5. Albumin fractions showed a constant EC ranging from 100 to 111 m^2^/g protein. TG has a high EC at pH 3, that is, 124 m^2^/g protein. This value decreased by about 40% at pH 5 and 7, in turn increasing at pH 9 to EC 135 m^2^/g protein (*P *<* *0.05)*. Dh*PI showed higher EC at pH 3 and 9, which ranged from 80 to 110 m^2^/g protein. On comparison with soy at pH 5.8 and 8.0, it was observed that *D. heterantha* had an EC of 74 and 105 m^2^/g protein, and the soy protein isolate had an EC of 47 and 78 m^2^/g protein, respectively (Liu et al. 2008).

The ES of each protein fraction was analyzed (Fig. 2B), in which glutelins demonstrated greatest stability (58 ± 11, 43.14 ± 14, and 33.38 ± 1.7 min at pH 5, 7, and 9, respectively) (*P < *0.05), followed by TGs, but only at pH 3 with an ES of 42 min (*P < *0.05). The remainder of the fractions had an EC of <10 min.

#### Gelling capacity

The effect on the pH of LGC (Table 5) reflected that albumin and TG showed a LGC (strong gel) of 80 and 100 g/kg at pH 5, respectively, while the glutelin fraction showed 90 g/kg of LGC at pH 3. Globulin II showed a LGC with strong gel at pH 9 with 100 g/kg. Globulin I and *Dh*PI showed weak gels with 70 g/kg protein at pH 7 and 90 g/kg protein at pH 9, respectively. This property is dependent on the pH (especially at alkaline pH) and protein concentration. The results agree with those of Lawal et al. (2005), who had researched albumin and globulin from African locust bean and found that gelation was pH dependent.

**Table 5:**
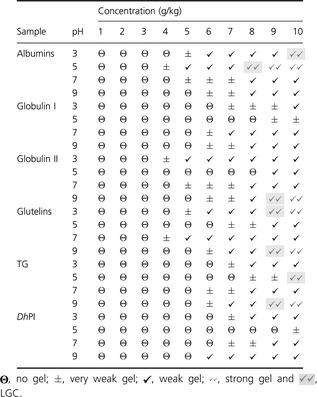
Least gelling concentration (LGC) of albumins, globulin I, globulin II, total globulins (TG), glutelin fraction, and *Ditaxis heterantha* protein isolate (*Dh*PI) at pH 3, 5, 7, and 9

Similarly, globulins from the Great Northern bean did not form a strong gel until reaching a concentration of 200 g/kg (w/v), but its isolate exhibited a LGC of 120 g/kg (Shate and Salunkhe 1981), indicating a lower LGC than *Dh*PI in the present study in which the protein concentration plays an important role. Several seed proteins were studied at concentrations up to 200 g/kg (w/v), while the proteins in the present study were analyzed at 100 g/kg, indicating that at higher protein concentrations, the results could show higher LGC.

## Conclusions

*Ditaxis heterantha* presented three main protein fractions: albumin, globulins, and glutelin. Nutritionally, *Dh*PI and globulin II fractions comply with the requirements of the Joint FAO/WHO/UNU for children and adult. Glutelins, TG, and globulins are thermostable fractions, are able to experience temperatures >100°C, and are important for use in technological processes. As regards functional properties, glutelins and globulins possessed EC and ES. Glutelins showed EC and FC with good stability. Gelling capacity was good in albumins and globulins. Protein fractionation showed that all protein fractions, with the exception of *Dh*PI, can be used as potential ingredients for food and feed supplements, thus providing an additional option for improving formulations in the food industry.

## Conflict of Interest

None declared.
